# Probiotics and NLRP3 mRNA inflammasome levels in women with endometriosis-related infertility undergoing assisted reproductive technologies

**DOI:** 10.25122/jml-2023-0056

**Published:** 2023-10

**Authors:** Oksana Valerianivna Bakun, Nataliia Sergiivna Voloshynovych, Kristina Victorivna Dyak, Valentyna Hryhorivna Ostapchuk, Halyna Danylivna Koval, Antonina Anatoliivna Piddubna, Simona Raluca Iacoban

**Affiliations:** 1 Obstetrics and Gynecology Department, Bukovinian State Medical University, Chernivtsi, Ukraine; 2 Department of Pediatrics and Medical Genetics, Bukovinian State Medical University, Chernivtsi, Ukraine; 3 Department of Clinical Immunology, Allergology and Endocrinology, Bukovinian State Medical University, Chernivtsi, Ukraine; 4 Department of Obstetrics and Gynecology, Polizu Clinical Hospital, Carol Davila University of Medicine and Pharmacy, Bucharest, Romania

**Keywords:** endometriosis, assisted reproductive technologies, inflammasome

## Abstract

This research was conducted at Bukovinian State Medical University and the Centre of Reproductive Medicine and included a total of 30 infertile women. The control group consisted of 10 women with tubal infertility resulting from a prior history of inflammation, who, following a comprehensive clinical and laboratory assessment, exhibited no other underlying health conditions and could be regarded as essentially healthy individuals. Participants in the control group ranged from 21 to 42 years, with an average age of 29.75 years. The control group did not receive the probiotic *Femina Probiz*. The main group, on the other hand, included 20 women diagnosed with external genital endometriosis who were undergoing assisted reproductive technologies. These patients were administered the probiotic *Femina Probiz* manufactured by Unic Biotech Ltd in India. They were instructed to take one tablet containing 10×10^9 *Lactobacillus* organisms twice daily for one month as part of their preparatory treatment before proceeding with assisted reproductive technologies. The levels of NLRP3 inflammasome were measured before and after this preparatory phase. The incidence of primary infertility was significantly higher in patients belonging to the main group.

## INTRODUCTION

Endometriosis has emerged as a pressing concern in contemporary gynecology due to its high prevalence and increasing incidence, affecting nearly one in every three women, becoming a topic of particular interest for both researchers and medical practitioners [1]. It is estimated that endometriosis affects over 10% of reproductive-age women globally, amounting to approximately 176 million women worldwide [1]. Understanding the pathogenesis and mechanisms of infertility associated with endometriosis remains a critical area of research. The same pathogenetic mechanisms responsible for infertility in endometriosis may also contribute to unsuccessful outcomes in in vitro fertilization (IVF) procedures [2].

Addressing endometriosis-associated infertility poses a significant challenge, as current therapeutic approaches primarily rely on surgical removal of endometrial lesions followed by hormonal treatments, which do not consistently demonstrate satisfactory effectiveness. The success of the most potent treatment approach, surgery, largely depends on the form and stage of the disease, the extent of ectopic tissue infiltration into the peritoneum, and the accompanying scar-dystrophic changes that occur during disease progression [2]. However, our understanding of the immunopathogenetic mechanisms driving endometriosis development, the potential impact of pharmacological interventions on clinical manifestations of endometriosis-related infertility, and strategies to enhance the efficacy of IVF through pathogenetically informed and optimized preparation remains limited [3, 4].

There is a possibility that effector pathways involving inflammasome activation may play a role in endometriosis pathogenesis. It is conceivable that inflammation could trigger progressive tissue damage, initiating the development of a chronic disease. A key cytokine in this context is interleukin-1 beta (IL-1β), which plays a pivotal role in regulating the adhesion and proliferation of endometrial cells [5, 6].

In one study, the influence of lactobacilli on the morphology of endometriosis lesions in an experimental model of endometriosis was examined [7]. The research found that treatment with lactobacilli led to a range of changes in the endometriosis lesions, suggesting the development of significant pathological processes and tissue disruption. This study aimed to assess messenger ribonucleic acid (mRNA) NLRP3 inflammasome levels in women experiencing infertility due to endometriosis who are undergoing assisted reproductive technologies with the addition of probiotics.

## MATERIAL AND METHODS

### Study location and design

This retrospective study was conducted at the Centre of Reproductive Medicine, Bukovinian State Medical University, Ukraine. The study design involved 30 female participants who were categorized into the following groups:

**The control group:** This group included 10 women with tubal infertility due to a previous history of inflammatory conditions. Following comprehensive clinical and laboratory evaluations, no additional health issues were identified, and they could be considered comparable to generally healthy women. These individuals, aged between 21 and 42, with an average age of 29.75 years, did not receive the *Femina Probiz* probiotic.

**The endometriosis group (main group)** included 20 women with external genital endometriosis who were undergoing assisted reproductive technologies. Patients in this main group were administered the *Femina Probiz* probiotic manufactured by Unic Biotech Ltd, India. They took one tablet containing 10×10^9 *Lactobacillus* twice daily for a month as part of their preparatory treatment before undergoing assisted reproductive technologies. NLRP3 inflammasome indices were assessed before and after this preparation.

### Measurement of NLRP3 inflammasome

To assess the expression of the NLRP3 inflammasome gene and determine the relative normalized expression of NLRP3 mRNA, we employed real-time reverse transcription polymerase chain reaction (RT-PCR). The study focused on mononuclear cells extracted from the whole blood of patients with endometriosis as the molecular genetic research subjects.

### Statistical analysis

Student's t-test was utilized to compare the two groups regarding quantitative variables, assuming normality and variance equality. Furthermore, to assess the diagnostic performance of quantitative variables in predicting categorical outcomes, a receiver operating characteristic (ROC) analysis was conducted. The optimal threshold value for the quantitative variable was determined using Youden's J statistic.

## RESULTS

The mean age of participants in the control group was 29.75±7.09 years. In contrast, the mean age of participants in the main group who received the probiotic was 30.65±2.04 years (p>0.05), indicating no statistically significant difference in age between the two groups. We assessed the infertility degree stratified by group (Table 1).

**Table 1:** Infertility degree among study groups

Variable	Categories	Group		p-value
		Control	Endometriosis	
Infertility degree	1	3 (30.0)	15 (75.0)	0.045*
	2	7 (70.0)	5 (25.0)	

* differences are statistically significant (p<0.05)

**Figure 1:**
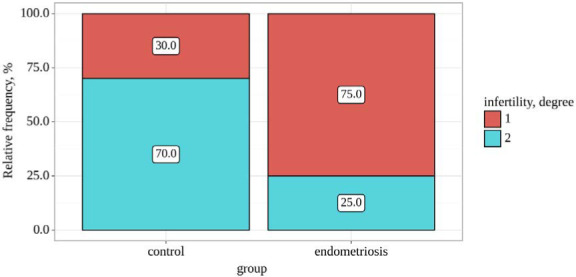
Infertility degree among study groups

Significant differences were observed regarding infertility degree between the control and endometriosis groups (p=0.045), as determined by Fisher's exact test (Table 1 and Figure 1). The endometriosis group showed a higher prevalence of primary infertility compared to the control group. The odds of experiencing degree 2 infertility were 7.000 times lower in the endometriosis group compared to the control group, indicating a statistically significant difference (OR=0.143; 95% CI: 0.026–0.774).

**Figure 2:**
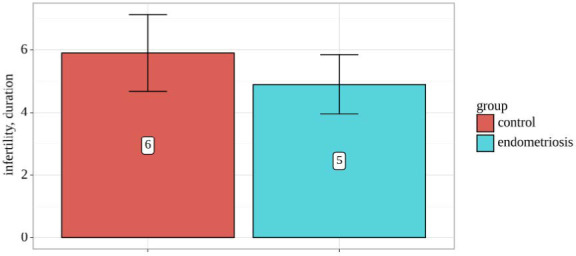
Duration of infertility among groups

Furthermore, we analyzed the duration of infertility among study groups (Table 2 and Figure 2). No statistically significant differences were observed in infertility duration (p=0.193), as assessed using Student's t-test, indicating similar infertility duration in both groups.

**Table 2:** Duration of infertility among groups

Group	Duration of infertility			p-value
	M±SD	95% CI	N	
Control	6±2	5-7	10	0.193
Endometriosis	5±2	4-6	20	

**Figure 3:**
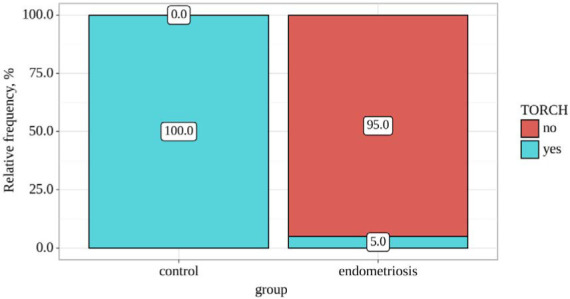
TORCH prevalence among groups

Table 3 and Figure 3 presents the analysis of TORCH infection based on the groups. Significant statistical differences were identified between the groups concerning TORCH infection (p<0.001), determined by Fisher's exact test. The endometriosis group had a higher prevalence of TORCH infection.

**Table 3:** TORCH prevalence among study groups

Variable	Categories	Group		p-value
		Control	Endometriosis	
TORCH	No	0 (0.0)	19 (95.0)	<0.001*
	Yes	10 (100.0)	1 (5.0)	

* differences are statistically significant (p<0.05)

**Figure 4:**
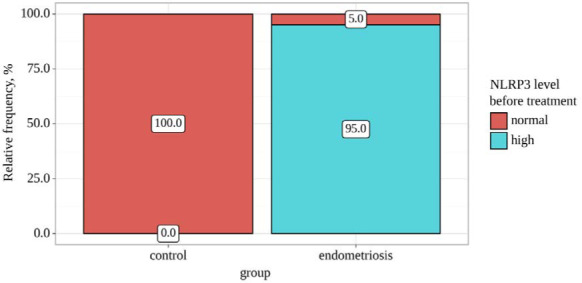
Analysis of NLRP3 level before treatment

The data analysis revealed statistically significant differences in NLRP3 levels before treatment, depending on the group (p<0.001), using Fisher's exact test. Table 4 illustrates the significant differences in NLRP3 levels before treatment between the control and endometriosis groups. In the control group, all participants (100.0%) had normal NLRP3 levels, while in the endometriosis group, the majority (95.0%) exhibited high NLRP3 levels, with only 5.0% showing normal levels (Figure 4). These differences were highly significant (p<0.001), indicating a strong relationship between elevated NLRP3 levels and endometriosis.

**Table 4:** NLRP3 levels before treatment

Variable	Categories	Group		p-value
		Control	Endometriosis	
NLRP3 level before treatment	Normal	10 (100.0)	1 (5.0)	<0.001*
	High	0 (0.0)	19 (95.0)	

* differences are statistically significant (p<0.05)

**Figure 5:**
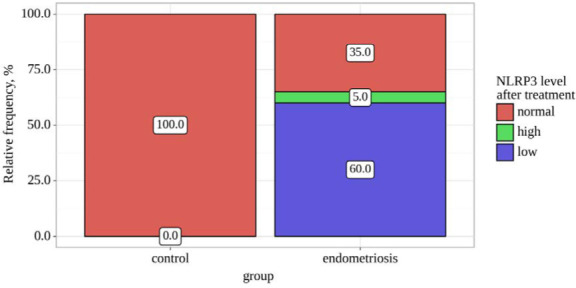
NLRP3 levels after treatment

Statistically significant differences in NLRP3 levels after treatment were identified when comparing the control and endometriosis groups (p=0.003) using Pearson's chi-square test. NLRP3 levels were assessed before and after treatment in the control and endometriosis groups. Table 5 and Figure 5 presents the distribution of NLRP3 levels after treatment, highlighting statistically significant differences between the two groups (p<0.05). Specifically, in the control group, all participants displayed normal NLRP3 levels. In contrast, within the endometriosis group, NLRP3 levels varied, with the majority falling into the low category.

**Table 5:** NLRP3 levels after treatment

Variable	Categories	Group		p-value
		Control	Endometriosis	
NLRP3 level after treatment	Normal	10 (100.0)	7 (35.0)	0.003*
	High	0 (0.0)	1 (5.0)	
	Low	0 (0.0)	12 (60.0)	

* differences are statistically significant (p<0.05)

**Figure 6:**
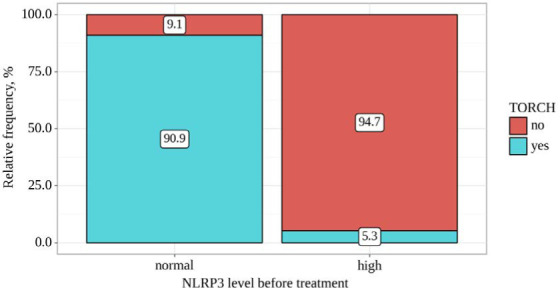
TORCH infections and NLRP3 levels before treatment

We analyzed TORCH infection status, considering the NLRP3 levels before treatment (Table 6 and Figure 6). Statistically significant differences were observed when comparing the presence of TORCH infections with respect to NLRP3 levels before treatment (p<0.001) (applied method: Fisher's exact test). Moreover, the odds of TORCH infections were approximately 180,000 times lower in individuals with high NLRP3 levels before treatment compared to those with normal NLRP3 levels before treatment (OR=0.006; 95% CI: 0.000 – 0.099).

**Table 6:** TORCH infections and NLRP3 levels before treatment

Variable	Categories	NLRP3 level before treatment		p-value
		Normal	High	
TORCH	No	1 (9.1)	18 (94.7)	<0.001*
	Yes	10 (90.9)	1 (5.3)	

* differences are statistically significant (p<0.05)

**Figure 7:**
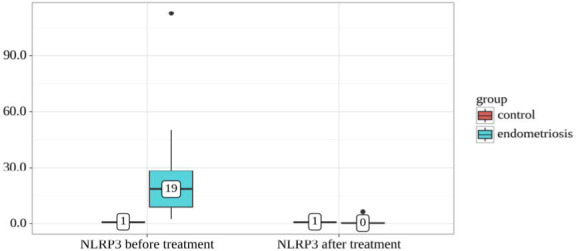
NLRP3 levels before and after treatment based on group

We conducted an analysis of NLRP3 dynamics before and after treatment, stratified by group (Table 7 and Figure 7). Statistically significant differences were observed when comparing NLRP3 levels before treatment between the control and endometriosis groups (p<0.001, Mann-Whitney U-test). Similarly, significant differences were found in NLRP3 levels after treatment (p=0.002, Mann-Whitney U-test). The analysis did not show statistically significant changes in the control group (Wilcoxon test). In contrast, in the endometriosis group, statistically significant changes in NLRP3 levels were observed before and after treatment (p<0.001, Wilcoxon test).

**Table 7:** NLRP3 levels before and after treatment based on group

Group	Follow-up periods				p-value
	NLRP3 before treatment		NLRP3 after treatment		
	Me	Q_1_–Q_3_	Me	Q_1_–Q_3_	
Control	1 (n=10)	1-1	1 (n=10)	1-1	–
Endometriosis	19 (n=20)	9-28	0 (n=20)	0-1	<0.001*
p-value	<0.001*		0.002*		–

* differences are statistically significant (p<0.05)

**Figure 8:**
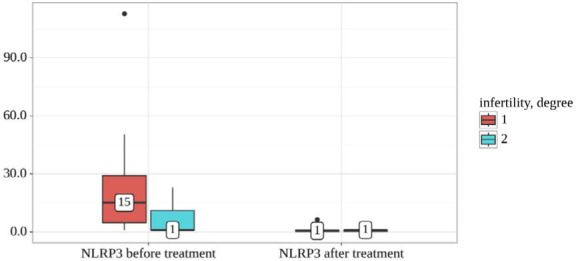
NLRP3 levels before and after treatment based on infertility degree

We analyzed NLRP3 dynamics before and after treatment, considering different infertility degrees (Table 8 and Figure 8). Statistically significant distinctions were observed when examining NLRP3 levels before treatment between the two infertility degree groups (p=0.013, Mann-Whitney U-test). Conversely, the analysis indicated no statistically significant differences when assessing the NLRP3 variable after the treatment period, with a p-value of 0.846 (employing the Mann-Whitney U-test). Furthermore, the analysis demonstrated statistically significant alterations in the first group, with a p-value of less than 0.001 (utilizing the Wilcoxon test). Similarly, there were statistically significant changes in the second group, with a p-value of 0.043 (employing the Wilcoxon test).

**Table 8:** NLRP3 levels before and after treatment based on infertility degree

Infertility degree	Follow-up periods				p-value
	NLRP3 before treatment		NLRP3 after treatment		
	Me	Q_1_– Q_3_	Me	Q_1_–Q_3_	
1	15 (n=18)	5-29	1 (n=18)	0-1	<0.001*
2	1 (n=12)	1-11	1 (n=12)	0-1	0.043*
p-value	0.013*		0.846		–

* differences are statistically significant (p<0.05)

**Figure 9:**
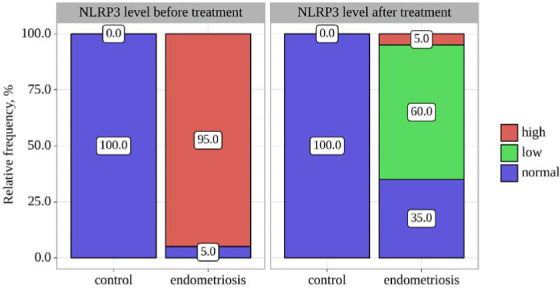
NLRP3 levels before and after treatment with group comparison

Significant statistical distinctions were identified when assessing the NLRP3 variable before the treatment period, with a p-value of less than 0.001 (utilizing Fisher's exact test) (Table 9 and Figure 9). Similarly, significant statistical differences were observed when evaluating the NLRP3 variable after treatment, with a p-value of 0.003 (employing Pearson's chi-square test). However, the analysis did not reveal statistically significant alterations in the endometriosis group, with a p-value of 0.157 (utilizing the Wilcoxon test).

**Table 9:** NLRP3 levels before and after treatment with group comparisons

Group	Variables	Follow-up periods				p-value
		NLRP3 level before treatment		NLRP3 level after treatment		
		Abs.	%	Abs.	%	
Control	High	0	0.0	0	0.0	–
	Low	0	0.0	0	0.0	
	Normal	10	100.0	10	100.0	
Endometriosis	High	19	95.0	1	5.0	0.157
	Low	0	0.0	12	60.0	
	Normal	1	5.0	7	35.0	
p-value		< 0.001*		0.003*		–

* differences are statistically significant (p<0.05)

## DISCUSSION

Endometriosis represents a global challenge in contemporary gynecology, garnering significant attention from the scientific community. It stands as one of the most prevalent conditions affecting women, often leading to fertility problems and a reduced quality of life. Approximately 1 in 10 women of reproductive age worldwide is estimated to experience endometriosis. This condition is diagnosed in 40-50% of women grappling with infertility and 70-90% of women experiencing chronic pelvic pain [8].

Despite the escalating number of scientific and clinical investigations into various aspects of endometriosis, many questions regarding its diagnosis, prevalence, course characteristics, and treatment remain contentious. The cost of treating endometriosis is on par with the expenses incurred for treating conditions such as Crohn's disease, type 2 diabetes, and rheumatoid arthritis [9]. Consequently, endometriosis remains a condition in the 21^st^ century without a universally accepted consensus on its root causes and etiological factors. Profound clinical symptoms, limitations in non-invasive diagnostic techniques, and the absence of a comprehensive treatment that guarantees freedom from relapse after therapy compel scientists and medical professionals to deepen their understanding and seek solutions for this pathology [10].

The specific mechanisms responsible for triggering the NLRP3 inflammasome remain incompletely understood. Possible processes encompass variations in intracellular calcium levels, lysosomal damage, mitochondrial impairment, potassium ion efflux, and the generation of reactive oxygen species (ROS) [11-15]. The NLRP3 inflammasome orchestrates the assembly of an inflammatory complex, bridging upstream NLRP3 and downstream caspase-1. Overactivation of the NLRP3 inflammasome leads to excessive production of inflammatory factors, resulting in pyroptosis and the onset of specific diseases. Endometriosis, a prevalent gynecological ailment, affects approximately 10-15% of women of childbearing age and 30% of women grappling with infertility [16, 17]. Recent research has proposed that immune factors play pivotal roles in endometriosis pathogenesis. Women with endometriosis often exhibit comorbidities such as hypothyroidism, susceptibility to vaginal candidiasis, autoimmune disorders, fibromyalgia, chronic fatigue syndrome, headaches, joint and muscle pain, asthma, and allergies. Peritoneal fluid samples from individuals with endometriosis indicate irregularly activated macrophages and natural killer cells that disrupt the recognition and clearance of endometrial cells. Inflammation is closely intertwined with endometriosis onset, representing a key pathophysiological foundation for the condition [18, 19]. The aberrant activation of inflammasomes, especially the NLRP3 inflammasome, is closely linked to the development of endometriosis. The NLRP3 inflammasome, a critical component of inflammasomes, functions as a significant inflammatory mediator in inflammatory responses [20, 21]. Investigations have revealed that reducing NLRP3 levels inhibits the production of inflammatory cytokines, indicating the potentially significant role of the NLRP3 inflammasome in endometriosis pathogenesis [19, 22].

However, many research endeavors have yet to fully elucidate the mechanisms of pathogenesis, non-invasive diagnostic methods, and the mechanisms underlying the potential suppression of the NLRP3 inflammasome through probiotic use. Furthermore, our study explores the impact of lactobacilli on the tissue of endometriotic lesions in experimental models of endometriosis. We have observed a range of changes in the endometriotic lesions after lactobacilli treatment, indicating significant development of pathological processes and destruction of these lesions [7]. The level of NLRP3 inflammasome from another study is shown in Table 10 [23].

**Table 10:** The level of NLRP3 inflammasome

Study groups	n	Expression of NLRP3 inflammasome level	p-value
Endometriosis	20	24.43	<0.05
Control	10	0.54	<0.05

The reduction in the expression level of the NLRP3 inflammasome observed in the control group of patients is noteworthy, as these individuals are essentially healthy women. Upon analyzing the data presented in Table 11, we can observe that the main group comprises women with endometriosis who underwent our proposed preparatory regimen for assisted reproductive technologies, including the incorporation of probiotics. The control group, on the other hand, underwent preparation for assisted reproductive technologies without the addition of probiotics. In the main group, the expression of NLRP3 inflammasome was initially measured at 24.43, which is significantly higher than the expression level after preparation, which decreased to 0.70. In contrast, in the control group, the expression of NLRP3 inflammasome was 0.54. The expression of NLRP3 inflammasome in patients increased more than 34-fold before preparation compared to after preparation, emphasizing a substantial reduction in NLRP3 inflammasome levels after probiotic use. This decrease underscores the effectiveness of probiotics and their potential utility in the preparation regimen for assisted reproductive technologies [23].

**Table 11:** The level of NLRP3 inflammasome before and after preparation

Group	Expression of Nlrp-3 inflammasome level		p-value
	Before preparation (treatment)	After preparation (treatment)	
Endometriosis	24.43	0.70	<0.05
Control	0.54	-	-
p-value	<0.05	-	

Furthermore, our study explored the impact of lactobacilli on the tissue of endometriotic lesions in experimental models of endometriosis. We have observed a range of changes in the endometriotic lesions after lactobacilli treatment, indicating significant development of pathological processes and destruction of these lesions [7].

## CONCLUSION

The remarkably elevated mRNA expression of the NLRP3 inflammasome suggests a close association between endometriosis pathogenesis and inflammation. Incorporating probiotics as part of a comprehensive preparation regimen before assisted reproductive technologies leads to a notable enhancement in patient well-being and a substantial reduction in mRNA expression of the NLRP3 inflammasome. Consequently, we strongly endorse the utilization of our preparation, which includes probiotics, in clinical practice.
